# Highly-Sensitive Thin Film THz Detector Based on Edge Metal-Semiconductor-Metal Junction

**DOI:** 10.1038/s41598-017-16923-z

**Published:** 2017-12-04

**Authors:** Youngeun Jeon, Sungchul Jung, Hanbyul Jin, Kyuhyung Mo, Kyung Rok Kim, Wook-Ki Park, Seong-Tae Han, Kibog Park

**Affiliations:** 1 R&D Center, SEMES, Hwaseong, Gyeonggi-Do, 18383 Republic of Korea; 2 Department of Physics, Ulsan National Institute of Science and Technology (UNIST), Ulsan, 44919 Republic of Korea; 3 School of Electrical and Computer Engineering, Ulsan National Institute of Science and Technology (UNIST), Ulsan, 44919 Republic of Korea; 4 Technology Convergence Center, Incheon Technopark, Incheon, 21999 Republic of Korea; 5 Korea Electrotechnology Research Institute, Changwon, Gyeongsangnam-Do, 51543 Republic of Korea

## Abstract

Terahertz (THz) detectors have been extensively studied for various applications such as security, wireless communication, and medical imaging. In case of metal-insulator-metal (MIM) tunnel junction THz detector, a small junction area is desirable because the detector response time can be shortened by reducing it. An edge metal-semiconductor-metal (EMSM) junction has been developed with a small junction area controlled precisely by the thicknesses of metal and semiconductor films. The voltage response of the EMSM THz detector shows the clear dependence on the polarization angle of incident THz wave and the responsivity is found to be very high (~2,169 *V*/*W*) at 0.4 THz without any antenna and signal amplifier. The EMSM junction structure can be a new and efficient way of fabricating the nonlinear device THz detector with high cut-off frequency relying on extremely small junction area.

## Introduction

Semiconductor terahertz (THz) radiation detectors are being used increasingly in various areas such as airport security, detection of unwanted foreign materials in processed food, and non-destructive inspection of cables^1–6^. The THz detector technology is also relevant for the THz wireless communication, which has become an important area of research due to the need for more rapid data transmission^7–9^, since the detector technology can serve as a base for developing efficient receivers essential in communication systems. However, one of the biggest problems in the THz technology is that THz waves are absorbed and attenuated quite a lot by water molecules in the atmosphere^7,10^. The THz wave absorption by water molecules occurs widely for the frequencies belonging to the THz bandwidth. Nonetheless, there are THz frequencies at which the absorption by water molecules is reduced greatly so that the corresponding THz waves can propagate very long distances in the air^11–14^. This is the main reason why many of the researches for developing THz wave detectors are dedicated to several specific frequencies including 0.4 THz which was chosen for our work. Until now, the researches on THz detector have been performed primarily by using field effect transistors (FETs) or diodes^15–21^. Recently, the THz detector based on graphene field effect transistor (GFET) has been studied actively since the response time can be short due to its high carrier mobility in comparison with other semiconductor FET THz detectors including Si metal-oxide-semiconductor field effect transistor (MOSFET)^22–24^. The THz detectors based on the nonlinearity of device current-voltage(I-V) characteristics have also been studied, mainly based on Schottky barrier diode and metal-insulator-metal (MIM) tunnel junction^25–28^. For MIM junction THz detectors, the research focus has been the reduction of junction area which is a crucial determining factor of the detector response time and cut-off frequency^20^. The most common strategy for reducing the junction area of MIM junction is to use electron beam lithography process guaranteeing nanometer-scale device structures. In this study, we devised and fabricated so-called Edge Metal-Semiconductor-Metal (EMSM) lateral junction which shows the electrical characteristics very similar to the conventional MIM junctions^29^. In our EMSM structure, the junction area can be controlled precisely with the thicknesses of metal and semiconductor films. Our work proposes a new and efficient way of fabricating the nonlinear device THz detector with high cut-off frequency by using substantially simplified manufacturing processes.

## Results

### EMSM junction structure

**Figure 1:**
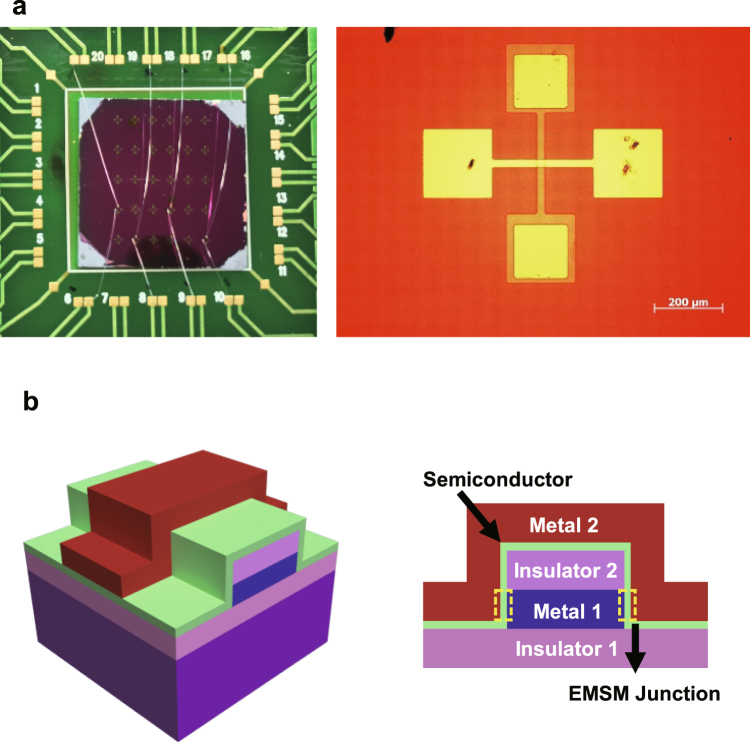
Structure of EMSM junction THz detector. (**a**) The optical microscope image of EMSM THz detector array on a PCB (Printed Circuit Board) and the zoom-in of EMSM junction. (**b**) The 3-dimensional schematic of EMSM junction structure and its cross-sectional schematic view. The yellow dashed boxes indicate the EMSM junction.

Figure 1a shows the optical microscope image of the EMSM THz detector array and the zoom-in of one detector with a crossbar structure. Figure 1b illustrates the 3-dimensional schematic of EMSM lateral junction and its cross-sectional view across the junction. The EMSM junction was fabricated on a SiO_2_/Si substrate and its material composition is as follows: Ni for “Metal 1” (60 nm), SiO_2_ for “Insulator 2” (30 nm), intrinsic SiC for “Semiconductor” (10 nm), and Ni/Au for “Metal 2” (30 nm/80 nm). We here note that the SiO_2_ layer of the substrate is “Insulator 1” in Fig. 1b. The role of Insulator 2 is to block the current flow from the Metal 2 into the upper side of Metal 1 and to enforce the current flow only through the edge sides of Metal 1 (yellow dashed rectangles). The intrinsic SiC layer can take a role of a thin insulator between the Metal 1 and Metal 2 because the amorphous SiC (a-SiC) is a wide bandgap semiconductor. Therefore, the EMSM lateral junction can behave quite similarly to a MIM junction in terms of its electrical characteristics. Since the EMSM junction is formed laterally on the side walls of Metal 1, the junction area can be controlled minutely with the Metal 1 and semiconductor film thicknesses. Hence, the junction area can also be drastically reduced by decreasing the film thicknesses down to nanometer scale. As it is well known, MIM junction THz detectors utilize the nonlinear I-V characteristics originating from quantum mechanical tunneling. Since the tunnel junction itself shows very fast response, the THz detector response time is mainly determined by the RC time delay of the entire detector circuit^20^. Therefore, the junction capacitance should be reduced to get better detector responses. Since the MIM junction can be considered as a parallel plate capacitor, its capacitance is given as *C* = *ε*_0_ *ε*_*r*_ (*A*/*d*). Here, *ε*_0_ is the vacuum permittivity, *ε*_*r*_ the dielectric constant of insulating layer, *A* the junction area, and *d* the thickness of insulating layer. Thus, most of researches have been devoted to minimizing the junction area for making the capacitance of MIM junction as small as possible^20,21^. In case of the conventional vertical MIM junction, the reduction of junction area enough for achieving THz response is done normally by using electron beam lithography. Meanwhile, the junction area of our EMSM structure can become quite small just by controlling the film thicknesses of Metal 1 and SiC layers as can be noticed in Fig. 1b. The thicknesses of those two films can be reduced down to several nanometers with a few angstrom precision with well-maintained film continuity. The lateral length of EMSM junction along the edge direction is ~20 μm and the thicknesses of Metal 1 and SiC layers are ~60 nm and ~10 nm each. Then, the junction area, which is the product of the lateral length along the edge direction and the thickness difference between Metal 1 and SiC layers, is calculated to be ~10^−12^ m^2^. The junction area of our EMSM structure is expected to be reduced further down to ~10^−14^ m^2^ without using any nanometer scale lithography process such as electron beam lithography. This can be achieved with the film thickness of tens of nm (~10^−8^ m) and the junction lateral length of a few μm (~10^6^ m) which can be patterned by using the conventional ultraviolet photolithography.

### Theory for junction current-voltage characteristics

**Figure 2:**
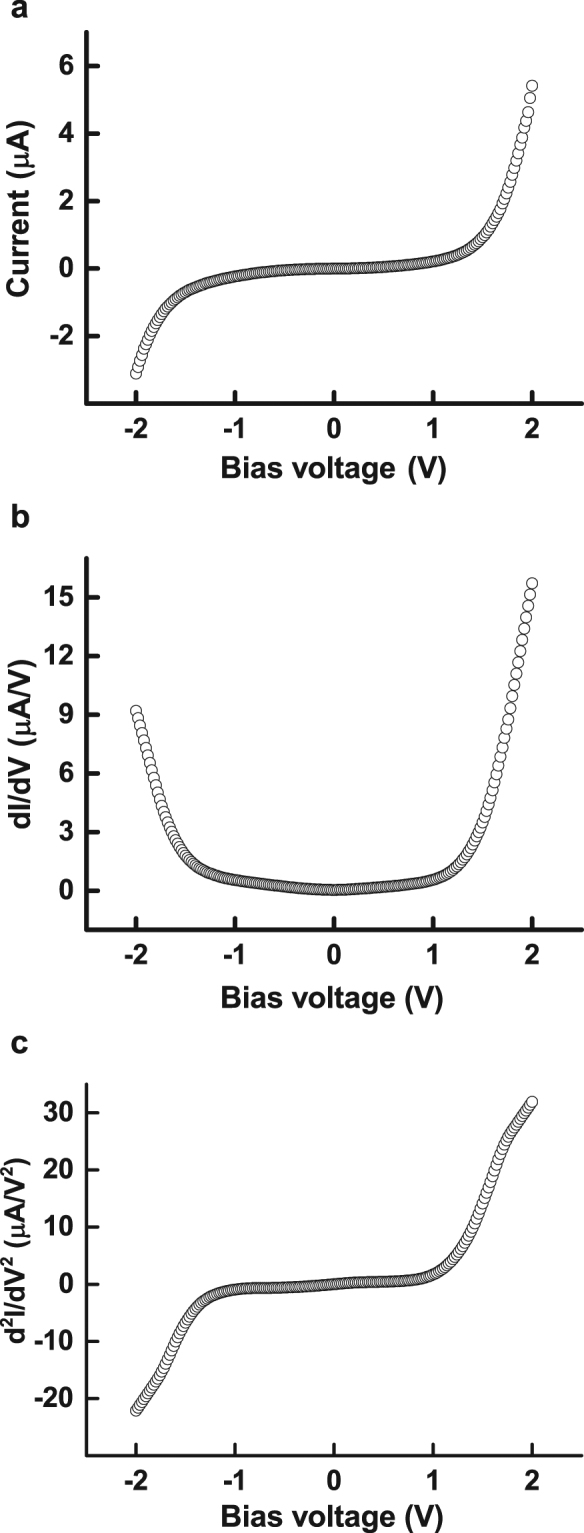
The electrical properties of EMSM junction. (**a**) Current-voltage curve and (**b**) first and (**c**) second derivatives of current-voltage curve.

The current-voltage (I-V) curve and its first and second derivatives of EMSM junction are presented in Fig. 2. Here, we note that the first and second derivatives were obtained by applying the Savitzky-Golay smoothing procedure to the measured I-V curve. As shown in Fig. 2a, the I-V curve shows a nonlinear behavior. Both first and second derivatives of I-V curve (Fig. 2b and c) are found to increase as the applied bias voltage increases, implying that the I-V curve becomes more and more nonlinear with the bias voltage. This nonlinear aspect of the I-V curve of EMSM junction is quite similar to that of MIM junction, which is one of the most crucial parameters determining the DC voltage response for THz wave detection. The nonlinear I-V characteristics are known to produce rectified DC voltage responses to external oscillating electric fields^30,31^. The rectified DC voltage (*V*_*rec*_) induced between the two sides of EMSM junction with a DC bias *V*_*b*_ applied is then expressed to be,

1 $${V}_{rec}({V}_{b})=\frac{1}{4}\frac{I^{\prime\prime} ({V}_{b})}{I^{\prime} ({V}_{b})}{V}_{0}^{2}.$$

Here, *V*_0_ is the magnitude of the AC voltage applied across the junction due to an external oscillating electric field. *I*′ and *I*″ are the first and second derivatives of I-V curve, respectively. More detailed derivation procedures of Eq. 1 are included in Supplementary Information. As noticed in Eq. 1, the rectified voltage is proportional to the square of AC voltage magnitude and the ratio of the second derivative (*I*″) to the first derivative (*I*′) of the I-V curve. The first and second derivatives are the junction characteristics and the AC voltage magnitude is related to the properties of incoming oscillating electric field. Therefore, the voltage response of THz detector can vary depending on the incoming THz wave power and the polarization direction of associated electric field in addition to the I-V characteristics of junction itself.

### Experimental set-up for characterizing THz detector

**Figure 3:**
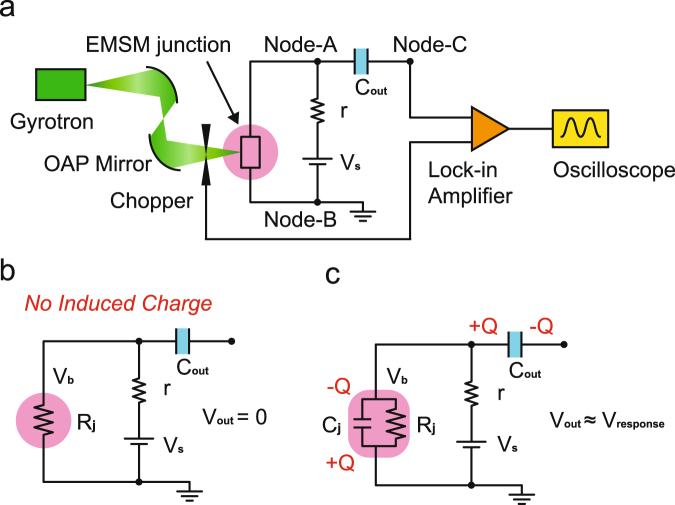
Schematic diagram of THz detecting system. (**a**) Configuration of THz detecting measurement set-up where the chopper frequency for lock-in detection was 200 Hz. The equivalent circuit diagram (**b**) *without* and (**c**) *with* the incoming THz wave.

As shown in Fig. 3a, the 0.4 THz beam was generated from a continuous wave (CW) sub-THz gyrotron. As described previously, THz waves attenuate quite quickly in the air due to the water molecules contained in it except for several specific frequencies. 0.4 THz is one of the frequencies that can travel relatively long distance in the air with the fairly small attenuation by water molecules^7,8^. Hence, we chose the 0.4 THz beam for evaluating the performance of our EMSM junction detector. The THz beam emitted from the gyrotron propagates to the first off-axis parabolic (OAP) mirror and reflects toward the second OAP mirror. After being reflected from the second mirror, the THz beam goes through the chopper and is focused on the EMSM junction detector connected to the read-out circuit sketched in Fig. 3a. In Fig. 3, *C*_*j*_ is the capacitance of EMSM junction, *R*_*j*_ the resistance of EMSM junction, *V*_*s*_ the DC voltage source, and *r* the resistance connected in series to the voltage source. The chopper driving signal with the frequency of 200 Hz is fed into the reference input of lock-in amplifier to perform the narrow-band measurements for the detector response, which can improve the signal-to-noise ratio of measurement significantly. In Fig. 3b, the equivalent circuit in the absence of the incoming THz wave is shown. Without any external AC voltage, the output voltage *V*_*out*_ will be zero because the EMSM junction works just as a simple resistor, resulting in no induced charges at both ends of the junction. However, when the THz wave hits the EMSM junction, a rectified voltage will be induced between two electrodes as described in the previous section. In principle, the rectified voltage is due to the net charge transfer across the EMSM junction relying on the nonlinear junction I-V characteristics, which causes the accumulation of opposite-sign charges on both sides of junction. The equivalent circuit revealing the charge accumulation on both sides of EMSM junction can be established to have the junction capacitance *C*_*j*_ connected in parallel to the junction resistance *R*_*j*_ (Fig. 3c). With the THz wave hitting the EMSM junction shown in Fig. 3c, a net negative charge (−*Q*) will be induced on the upper side of EMSM junction while the same magnitude of positive charge (+*Q*) will be induced on the lower side. In order to compensate these accumulated charges, a positive charge +*Q* and the same magnitude of negative charge −*Q* will be induced on the left and right side of the output-terminal capacitor *C*_*out*_, respectively. Here, the EMSM junction and the *C*_*out*_ are connected in series, meaning that the output voltage (*V*_*out*_) is determined by the sum of the voltage across *C*_*j*_ and *C*_*out*_. However, the voltage across *C*_*j*_ is much larger than that across *C*_*out*_ because *C*_*j*_ has much smaller capacitance (~8.14 fF) compared to *C*_*out*_ (~100 nF). Consequently, *V*_*out*_ measured between Node-B and Node-C is almost identical to the voltage across *C*_*j*_ which is just the voltage response of EMSM junction (*V*_*response*_) to the incident THz wave. Finally, the output voltage is amplified with a lock-in amplifier and the signal is read in an oscilloscope (Fig. 3a). Some more details for the lock-in measurement of the detector response are described in Supplementary Information.

### Voltage response of EMSM junction depending on THz beam power and polarization direction

**Figure 4:**
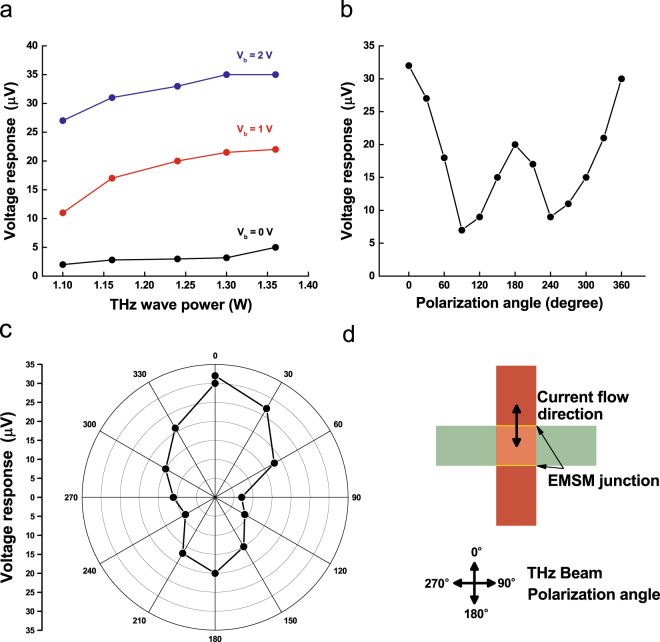
The voltage response of EMSM junction THz detector. (**a**) The voltage response as a function of incident THz beam power at 0.4 THz frequency for several different junction biases. (**b**) The line plot and (**c**) the polar contour plot of the voltage response for varying the THz wave polarization angle at 0.4 THz frequency and 2 V bias voltage. (**d**) The schematic view of the relative orientation between THz beam polarization angle and EMSM junction.

**Figure 5:**
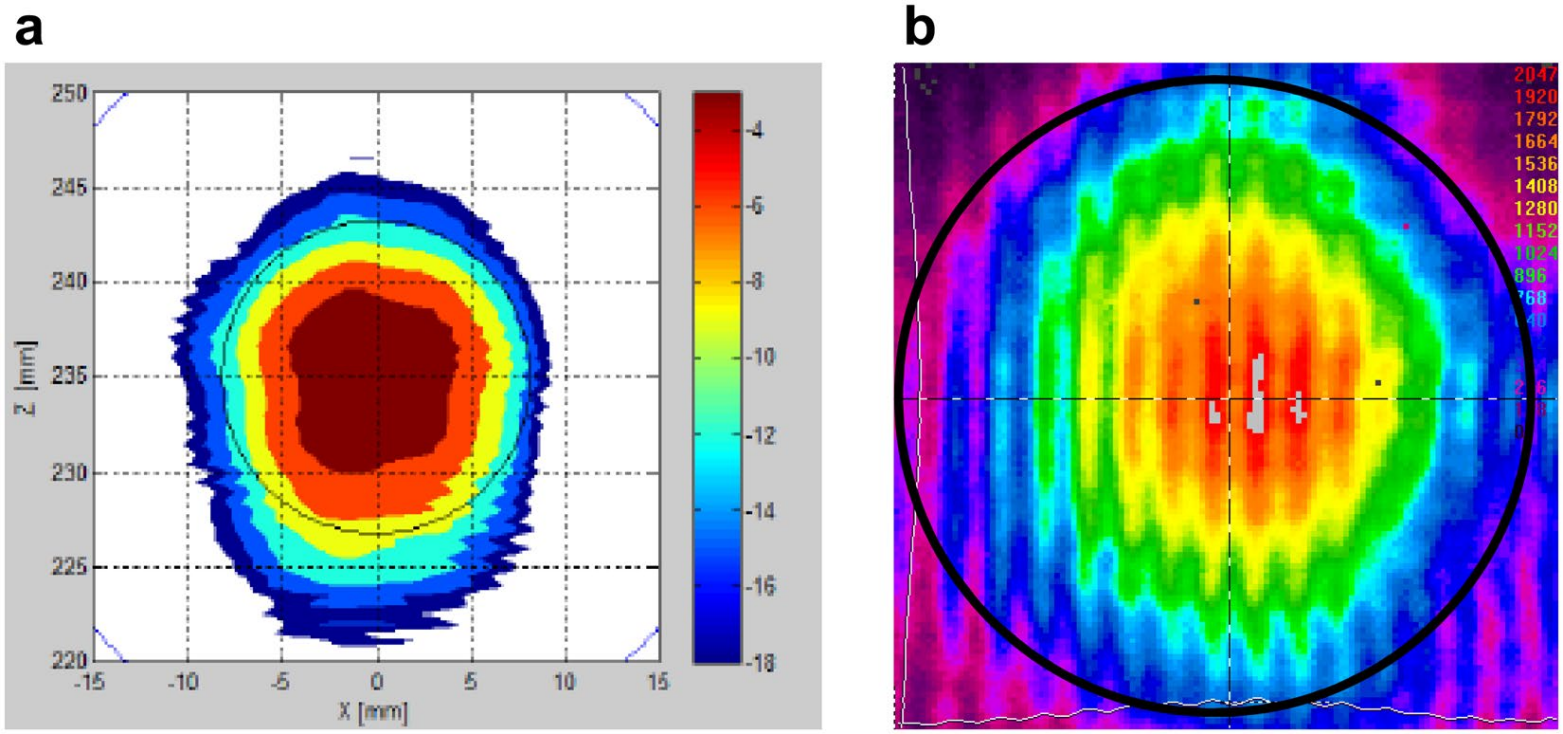
The spatial profile of the THz beam emitting from the gyrotron. (**a**) Simulated and (**b**) measured profiles of THz beam. For both (**a**,**b**), the THz rays falling into the area enclosed by the black circle were included to acquire the total power of incident THz beam.

Figure 4a shows the voltage response of EMSM junction as a function of THz beam power at the frequency of 0.4 THz. As expected from Eq. 1, the voltage response becomes larger as THz beam power increases. The voltage response increases also with *V*_*b*_ increasing due to the enhanced nonlinearity of I-V characteristics at higher applied biases (Fig. 2c). Figure 4b and c show the dependence of voltage response on the polarization direction of THz wave. The AC voltage induced across the EMSM junction due to the incident THz wave is regulated by the oscillating electric field direction of THz wave (Fig. 4d). When the electric field of incident THz wave is in the direction perpendicular to the SiC layer of EMSM junction, the magnitude of AC voltage is maximized (around 0 and 180 degrees in Fig. 4b and c). In contrast, the magnitude of AC voltage is minimized when the oscillating electric field direction is parallel to the SiC layer (around 90 and 270 degrees in Fig. 4b and c). In our measurements, the voltage response of EMSM junction was found to become maximized at the polarization angle of 0 and 180 degrees and minimized at 90 and 240 degrees. One thing to note here is that the voltage response of EMSM junction at 180 degree is somewhat weaker than that at 0 degree, which should be almost identical ideally. This is believed to be the combinatorial effect of the asymmetric distribution of THz beam power and the misalignment between the structural center of EMSM detector and the rotational axis of sample stage. The THz beam emitted from our gyrotron was diagnosed not to be so symmetric in its power and electric field strength as shown in Fig. 5. Figure 5a is the simulation result of THz beam power profile and Fig. 5b is the measured power profile. The black circles in Fig. 5a and b are the boundaries of the areas used for acquiring the total power of THz beam. The THz wave power in Fig. 4a is the total power inside the black circle in Fig. 5b. The 0 degree polarization direction of THz beam was set to be the one at which the voltage response of EMSM detector becomes maximized. Since the metal electrode crossing area where the two EMSM junctions reside at the two opposing edges is 20 μm × 20 μm, the perfect alignment of the crossing area center and the rotational axis of sample stage is not possible. Therefore, the maximum power region of THz beam could be shifted when the polarization direction changes from 0 to 180 degree. However, the overall dependence of the voltage response of EMSM detector on the polarization direction of THz beam matches well with the theoretical anticipation as described previously^20^.

### Responsivity of EMSM THz detector

The responsivity of EMSM THz detector is defined to be the voltage response of detector divided by the power of incident THz beam. When the total power of incident THz beam with its diameter of 15 mm is ~1.1 W (inside the black circle of Fig. 5b), the beam power per unit area is ~6,225 W/m^2^. Then, the THz beam power given to the EMSM junction is calculated to be ~1.25 × 10^−8^ W. With this THz beam power, the detector responsivity is estimated to be ~161 V/W at *V*_*b*_ = 0 V and it increases to ~2,169 V/W at *V*_*b*_ = 2 V. The responsivities of our EMSM detector are considered to be pretty high, especially if considering that no additional antenna and signal amplifier were involved. Just as comparison, the typical responsivities of recently-reported Schottky diode^32^ and semiconductor interband tunneling diode^33^ THz detectors with antenna structures incorporated are ~500 V/W and ~1,150 V/W, respectively, as described in Table 1. The recent THz detectors based on MOSFET^34^ and GFET^35^ with antenna structures, are reported to have responsivities of ~5,000 V/W and ~74 V/W each, also shown in Table 1. Based on the calculated responsivities, we can deduce the Noise-Equivalent Power (NEP) which represents the minimum detectable power per square root of frequency and can be calculated with the following relation.

2 $${\rm{NEP}}=\frac{{\rm{Noise}}\,{\rm{Spectral}}\,{\rm{Density}}\,(V/\sqrt{Hz})}{{\rm{Responsivity}}\,(V/W)}$$

**Table 1:** The comparison between EMSM junction THz detector and other types of THz detectors in terms of responsivity and NEP.

	Schottky Diode	Interband Tunneling Diode	MOSFET	GFET	This Work
Responsivity (V/W)	500^a^ (@ 0.4 THz)	1,150^b^ (@ 0.2 THz)	5,000^c^ (@ 0.3 THz)	74^d^ (@ 0.4 THz)	2,169 (@ 0.4 THz)
NEP ($pW/\sqrt{Hz}$)	5^a^ (@ 0.4 THz)	7^b^ (@ 0.2 THz)	10^c^ (@ 0.3 THz)	130^d^ (@ 0.4 THz)	14.9 (@ 0.4 THz)

The listed responsivities and NEPs are taken from ^a^ref.^32^, ^b^ref.^33^, ^c^ref.^34^, and ^d^ref.^35^.

Here, we just considered the Johnson noise^36^ as the noise spectral density, which is the electronic noise from the thermally agitated charges. The Johnson noise is described as $\sqrt{4{k}_{B}TR}$ where *k*_*B*_ is Boltzmann’s constant, *T* temperature, and *R* the junction resistance^37^. Because of the nonlinear I-V characteristics, the differential resistance has to be used to estimate the NEP of our EMSM junction at each bias. The differential resistance at *V*_*b*_ = 2 V is calculated to be ~63.7 kΩ. Then, the corresponding noise spectral density is ~32.4 $\,\mathrm{nV}/\sqrt{{\rm{Hz}}}$ at 300 K. In this case, the resulting NEP of our EMSM detector is estimated to be ~14.9 $\,\text{pW}/\sqrt{{\rm{Hz}}}$, which is comparable to the reported values for other types of THz detectors as shown in Table 1.

## Discussion

In summary, we fabricated the EMSM lateral junction composed all of thin films and confirmed that this junction work as a THz detector with high sensitivity. The lateral EMSM junction was found to possess nonlinear current-voltage characteristics similarly to the MIM junction, which is the essential aspect to be used as an electromagnetic wave detector. The junction area of EMSM junction can become quite small to achieve fast detector responses just by controlling the thicknesses of metal and semiconductor films without using nanoscale lithography processes. The voltage response of our EMSM junction detector was found to be proportional to the beam power of incident THz wave and show the clear dependence on the polarization angle of THz wave. The responsivity of EMSM junction detector was estimated to be very high (~2,169 V/W) at 0.4 THz with no antenna structure incorporated. In accordance with this high responsivity, the detector NEP is also estimated to be very low (~14.9 $\,\text{pW}/\sqrt{{\rm{Hz}}}$) when considering the Johnson noise spectral density. Based on its high performance together with cost-effective fabrication procedures utilizing the conventional thin film and photolithography processes, our EMSM junction can provide a reliable platform for developing high-performance multi-pixel THz detecting systems.

## Methods

### Detailed fabrication processes of EMSM structure

The EMSM structure was formed on a SiO_2_/Si substrate. First, the photoresist (AZ5214E) is spin-coated on the substrate and the coated photoresist was patterned by using the UV photolithography process with a mask aligner (MA6, SUSS MicroTec, Germany). After the photoresist patterning, “Metal 1” layer is deposited with e-beam evaporation (FC-2000, Temescal, USA) and “Insulator 2” layer is deposited by using RF magnetron sputtering (SRN-120D, SORONA, Korea) successively. Then, the lift-off process to form “Metal 1” and “Insulator 2” stacked lines is followed by using acetone. Next, a SiC thin film is deposited on the whole sample surface by using plasma enhanced chemical vapor deposition (Pilot PECVD Cluster System, ITS, Korea). On the deposited SiC film, “Metal 2” lines are formed by following the same procedures used for forming “Metal 1” and “Insulator 2” stacked lines. Finally, the SiC and “Insulator 2” layers on top of the “Metal 1” contact pad are etched by using reactive ion etching (Labstar, Top Technology Ltd, Korea) to make the connection between the contact pads of “Metal 1” and printed circuit board (PCB) where the read-out circuit elements are installed.

## Electronic supplementary material

Supplementary Information
