# Supplementary material for: Highly-Sensitive Thin Film THz Detector Based on Edge Metal-Semiconductor-Metal Junction
Source: Sci Rep. 2017 Dec 4;7:16830. doi: 10.1038/s41598-017-16923-z (PMC5715053; doi:10.1038/s41598-017-16923-z)
Supplement: Supplementary file 1 — Supplementary Information [file 41598_2017_16923_MOESM1_ESM.pdf]

Highly-Sensitive Thin Film THz Detector Based on Edge Metal-Semiconductor-Metal Junction

Youngeun Jeon[1]†, Sungchul Jung[2]†, Hanbyul Jin[3], Kyuhyung Mo[2], Kyung Rok Kim[3], Wook-Ki Park[4], Seong-Tae Han[5], and Kibog Park*[2,3]

[1]R&D Center, SEMES, Hwaseong, Gyeonggi-Do 44919, Republic of Korea

[2]Department of Physics, Ulsan National Institute of Science and Technology (UNIST), Ulsan 44919, Republic of Korea

[3]School of Electrical and Computer Engineering, Ulsan National Institute of Science and Technology (UNIST), Ulsan 44919, Republic of Korea

[4]Technology Convergence Center, Incheon Technopark, Incheon, 21999, Republic of Korea

[5]Korea Electrotechnology Research Institute, Changwon, Gyeongsangnam-Do 51543, Republic of Korea

Corresponding Author

Correspondence and requests for materials should be addressed to Kibog Park* (Email: kibogpark@unist.ac.kr)

Equal Contribution

†These authors contributed equally to this work.

Supplementary Figures

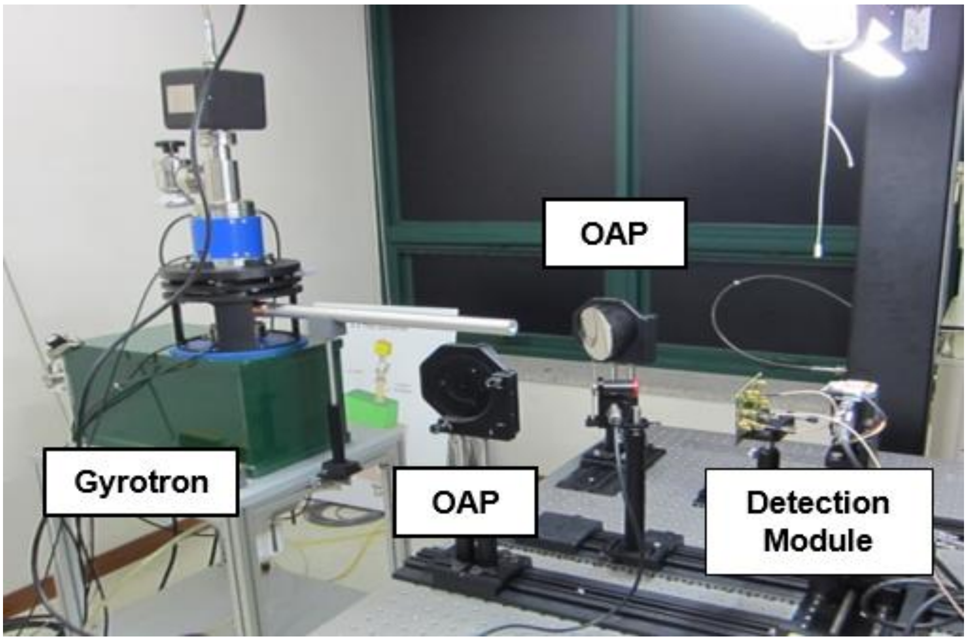

Supplementary Figure 1. The experimental set-up for characterizing THz detector

Derivation of rectified DC voltage for EMSM junction exposed to AC electric field

The total voltage across the EMSM junction is the sum of the applied DC bias and the AC voltage associated with an external oscillating electric field.

$$V = V_b + V(t) = V_b + V_0 \cos(\omega t) \tag{S.1}$$

Here, V_b is the applied DC bias, V_0 the magnitude of AC voltage, and ω the angular frequency of AC voltage. For this oscillating total voltage, the current can be described as a Taylor series like below.

$$I(V) = I(V_b) + \left.\frac{dI}{dV}\right|_{V=V_b} V(t) + \frac{1}{2}\left.\frac{d^2 I}{dV^2}\right|_{V=V_b} V^2(t) + \cdots \tag{S.2}$$

By considering that the AC voltage developed by the external oscillating electric field is normally small in its magnitude, the time-averaged total current through the EMSM junction can be approximated up to the most dominant second-order term as follows.

$$\begin{aligned} I_{avg} &\approx \frac{1}{2\pi}\int_{-\pi}^{\pi}\left[I(V_b) + \left.\frac{dI}{dV}\right|_{V=V_b} V(t) + \frac{1}{2}\left.\frac{d^2 I}{dV^2}\right|_{V=V_b} V^2(t) \right] d(\omega t) \\ &\approx I(V_b) + \frac{1}{4}\left.\frac{d^2 I}{dV^2}\right|_{V=V_b} V_0^2 \\ &\approx I(V_b) + I_{rec}\left(V_b\right), \qquad I_{rec}\left(V_b\right) = \frac{1}{4}\left.\frac{d^2 I}{dV^2}\right|_{V=V_b} V_0^2 \end{aligned} \tag{S.3}$$

Here, I_{rec} represents the rectified current occurring as the response to the external oscillating electric fields. Then, the additional DC voltage (V_{rec}) induced on both sides of EMSM junction due to the rectified current is now expressed to be

$$V_{rec} = \frac{I_{rec}\left(V_b\right)}{\left(dI/dV\right)_{V=V_b}} = \frac{1}{4}\frac{I''\left(V_b\right)}{I'\left(V_b\right)} V_0^2 . \tag{S.4}$$

Details of Measurement Scheme

In our experiments, the voltage response ($V_{response}$) of EMSM junction detector to the incident THz wave was measured with a low-noise amplifier adopting the lock-in technique. The rotational frequency of the chopper modulating the THz wave input periodically was 200 Hz which was used as the reference frequency (f_{ref}) of lock-in amplifier. Since the modulation frequency of the incident THz wave was 200 Hz, the voltage response of detector also had its frequency of 200 Hz. As usual in the standard lock-in technique, the total voltage signal (V_{out}) detected in the EMSM junction detector was multiplied by the sinusoidal reference voltage of f_{ref} synchronized to the chopper driving voltage in the lock-in amplifier and the product of them was integrated over time as shown in the relation below.

$$V_{response} = \frac{1}{T}\int_{t-T}^{t} \sin\left[2\pi f_{ref} \cdot s + \varphi\right] \cdot V_{out}(s)ds \qquad \text{(S.5)}$$

Here, t is an arbitrary time during measurements and $T = 1/f_{ref}$ is the modulation period of the incident THz wave. As noticeable clearly in Eq. 6, any voltage signal with its frequency different from the reference frequency is filtered as a noise and only the voltage signal with the reference frequency can survive. Hence, the actual voltage response of our EMSM junction detector to the incident THz wave can be measured with minimized noises.
